# Could Residents Adequately Assess the Severity of Skin Lesions in Mycosis Fungoides/Sézary Syndrome? Evaluation of Interrater Agreement and Interrater Reliability of mSWAT

**DOI:** 10.3390/jcm14010075

**Published:** 2024-12-27

**Authors:** Hanna Cisoń, Alina Jankowska-Konsur, Rafał Białynicki-Birula

**Affiliations:** 1 University Centre of General Dermatology and Oncodermatology, Wroclaw Medical University, 50-556 Wroclaw, Poland; alina.jankowska-konsur@umw.edu.pl; 2 Faculty of Medicine, Wroclaw University of Science and Technology, 50-370 Wroclaw, Poland; rafal.bialynicki-birula@pwr.edu.pl

**Keywords:** Mycosis fungoides/diagnostic imaging, Sezary syndrome/diagnostic imaging, ultrasonography

## Abstract

**Background/Objectives**: Cutaneous T-cell lymphoma (CTCL), including Mycosis fungoides (MF) and Sézary syndrome (SS), is a challenging-to-diagnose lymphoproliferative malignancy characterized by T-cell dysfunction and progressive cutaneous and extra cutaneous involvement. Disease severity assessment in CTCL is crucial for guiding treatment. This study aims to evaluate the interrater agreement and interrater reliability of mSWAT among dermatology residents and identify lesion types most prone to scoring variability. **Methods**: Sixteen dermatology residents with varied experience levels assessed 14 patients with confirmed MF/SS diagnoses. Using mSWAT, residents independently scored lesions severity on a standardized set of patient’s photos. The results were compared with reference mSWAT scores provided by an experienced clinician. Descriptive statistics and the Shapiro–Wilk test were applied to evaluate data distributions, while Student’s *t*-test assessed score deviations from reference values. Furthemore, we conducted a pilot the high frequency ultrasound (HFUS) study on a single patient, whose mSWAT score and photographs are also presented in the manuscript. **Results**: Significant discrepancies were observed in 64.29% of cases (9/14), with tumors and infiltrative lesions in erythrodermic SS patients posing particular scoring challenges. Misclassification of tumors as patches or plaques was a frequent issue, leading to underestimations in mSWAT scores. Residents’ assessments of infiltrative lesions were also notably inconsistent. **Conclusions**: This study highlights significant interobserver variability in mSWAT scoring among less experienced dermatology residents, particularly with tumor and erythrodermic lesions. Findings underscore the need for enhanced training and standardized scoring protocols to improve mSWAT reliability. Similar to other comparable indices, such as PASI, the mSWAT should be employed consistently by the same physician during each assessment to systematically monitor and evaluate the skin condition of a patient under observation. However, broader application requires the acquisition of sufficient experience. The study suggests the use of the HFUS as an objective method of assessment of the skin lesion infiltration in MF/SS patients.

## 1. Introduction

Cutaneous T-cell lymphoma (CTCL) represents a heterogeneous group of malignancies predominantly involving skin-tropic T-lymphocytes. In contrast to nodal non-Hodgkin lymphoma, which mainly originates from B-cells, approximately 75% of primary cutaneous lymphomas are of T-cell origin. Among these T-cell lymphomas, two-thirds are classified as Mycosis fungoides (MF) or as Sézary syndrome (SS) [1]. Therefore, we will focus only on MF and SS. MF generally manifests as slowly progressing cutaneous lesions. In advanced stages, MF may exhibit extracutaneous involvement, affecting lymph nodes, the bloodstream, and, less frequently, other organs [2]. On the other hand, SS is a leukemic variant of CTCL. It is characterized by erythroderma, palmoplantar keratoderma, pruritus, and peripheral blood involvement [3]. CTCL is marked by the progressive dysfunction of various components of the immune system. SS is thought to derive from thymic memory T cells whereas MF is hypothesized to originate from the malignant transformation of cutaneous-resident effector memory T cells. This differentiation implies that SS is a distinct entity from MF [4]. CTCL is a debilitating condition, strongly influencing patients’ quality of life. Initial management protocols for patients diagnosed with MF or SS typically include a comprehensive physical examination using the modified Severity Weighted Assessment Tool (mSWAT), histological exam, blood morphology analysis, and relevant imaging tools such as Computed Tomography (CT) and Fluorodeoxyglucose Positron Emission Tomography (FDG-PET) scans. Peripheral blood flow cytometry is generally advocated at all stages of MF whereas for intermediate or aggressive cutaneous lymphomas, it is recommended to perform a bone marrow biopsy and aspiration [5]. The immunophenotypic profile of MF and SS cells typically includes the expression of CD2, CD3, CD5, CD4, CCR4, TCR-beta, and CD45RO, with a lack of CD7 and CD26 markers [6]. Additionally, adverse prognostic factors in MF, independent of clinical stage, include older age (>60 years), large cell transformation, elevated lactate dehydrogenase (LDH) levels, and low CD30+ expression (<10%) [7,8]. The staging of MF and SS is based on the tumor-node-metastasis (TNM) classification system, which was first introduced in 1979 [9]. This system was revised and expanded in 2007 to include blood involvement, creating the tumor-node-metastasis-blood (TNMB) classification [10]. Another tool, the Severity-Weighted Assessment Tool (SWAT), originally introduced by Stevens et al. [11] in 2002, quantifies disease severity by severity of the affected skin in MF/SS, by assessing the percentage of total body surface area (%TBSA) changed by specific lesion types, including patch, plaque, tumor, and ulcer. This tool offers an objective and precise method to monitor changes in disease progression [12]. A subsequent modification, known as the modified SWAT (mSWAT), was developed to assign greater weight to tumors and to differentiate between erythrodermic and non-erythrodermic forms of the disease. The European Organization for Research and Treatment of Cancer (EORTC) endorses mSWAT as the preferred method for evaluating cutaneous involvement in MF and SS [13].

The objective of this investigation was to conduct and estimate a comparative analysis and assessment of the reliability and consistency of the scales employed for evaluating the severity of MF/SS among resident dermatologists.

## 2. Materials and Methods

**Figure 1:**
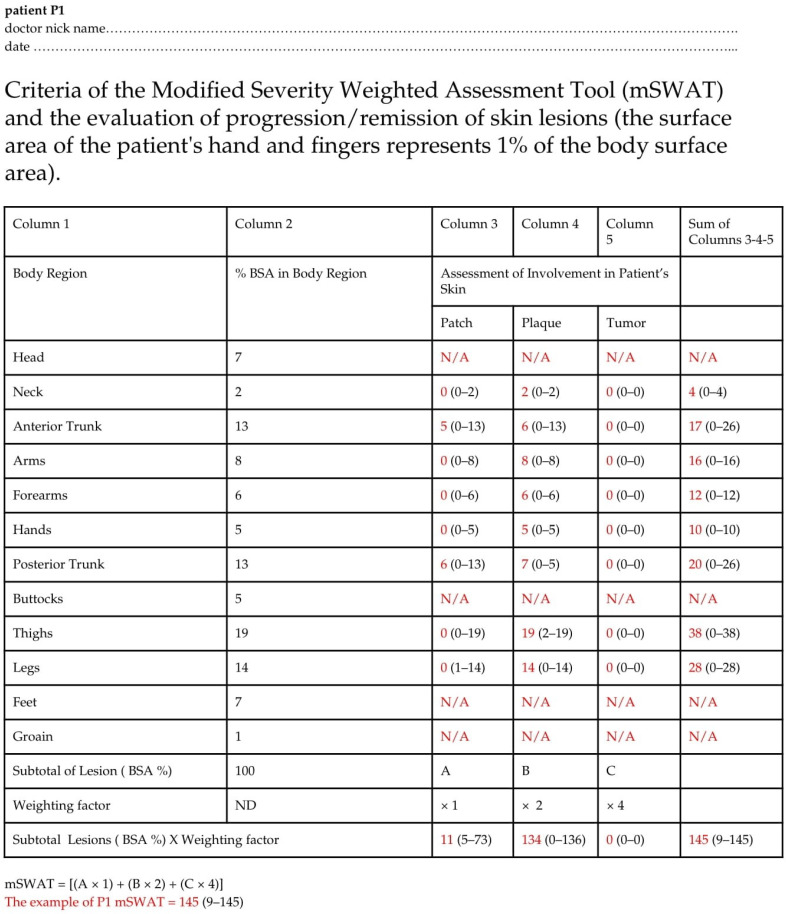
The mSWAT questionnaire, originally available in English, was utilized in its Polish version in our study to accommodate Polish-speaking residents. In the questionnaire is shown data of our patients (P1), (the English version) indicated in red color, and mSWAT = 145. The values in parentheses represent the range of assessments provided by the resident physicians. For legal reasons some body regions were not assessed: head, buttocks, groin. Feet were free of lesions in all patients. These cases were stated: N/A.

**Figure 2:**
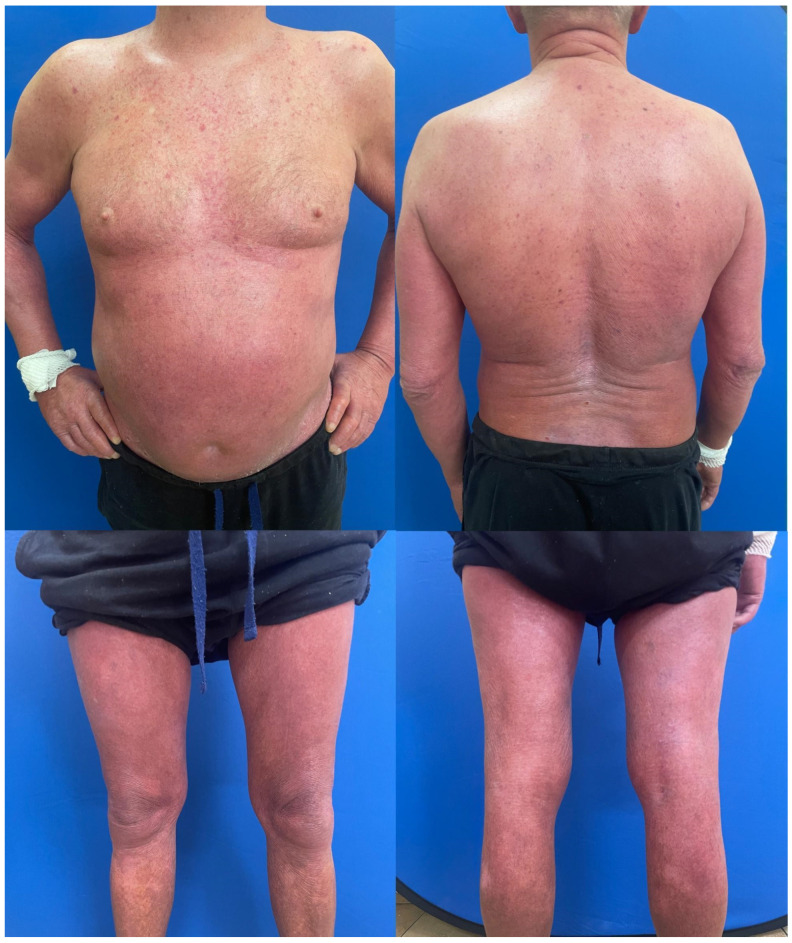
One of our patients (P1) exhibited the most pronounced changes in the assessment of infiltrative and erythematous lesions, with statistically significant differences observed in mSWAT evaluations. Reference mSWAT was 145, but mSWAT estimated by residents was from 9 to 110 (refer to Figure 3 and Figure 4 as well as Table 1 and Table 2).

**Figure 3:**
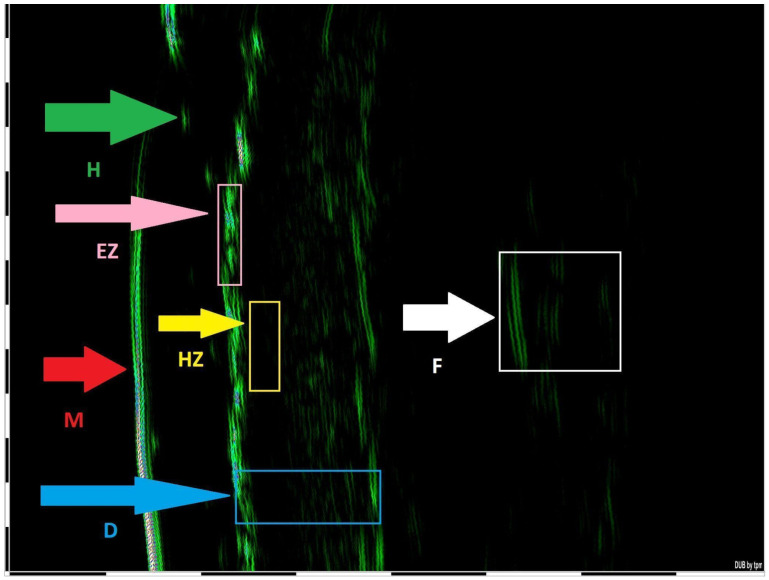
The HFUS (22.5 MHz) picture of the infiltration in our patients (P1)—the broad band hypoechogenic zone interpreted as infiltration under epidermis; terms in alphabetical order: **D**—dermis; **EZ**—entrance zone; **F**—fascia; **H**—hair; **HZ**—hypoechogenic zone; **M**—membrane.

In total, 16 dermatology resident doctors (13 females and 3 males) from the university department of dermatology in Poland were recruited as raters. Their ages ranged from 26 to 35 years, with dermatology experience between 1 and 5 years (mean: 2.9 ± 1.0 years). Of these, 6 were junior residents in the first half of their residency, while 10 were senior residents who had completed at least 2.5 years of their residency. All raters had prior clinical experience with CTCL patients through their work in the university hospital or outpatient settings and thus had knowledge of disease manifestations, pathophysiology, and treatment approaches. This study was based on the enrolment of 14 adult male patients with histological confirmed diagnoses of MF/SS, exhibiting varying degrees of disease severity and symptoms duration. All patients were recruited from the Department of Dermatology, Venereology, and Allergology, Wroclaw Medical University, Poland, and provided written informed consent to participate in the study. Scoring was conducted on a single day, during which all patients’ photos were evaluated by the dermatology residents using the modified Severity-Weighted Assessment Tool (mSWAT) to assess disease severity (Figure 1). Patients’ pictures were assigned identification numbers. Each patient’s pictures were independently examined by all residents, e.g., Figure 2. There were no time restrictions on the examinations and data were recorded on standardized scoring forms (Figure 1). The residents were instructed to work independently and avoid communication with one another until all evaluations were completed. Before the scoring session, the raters participated in a short training session for 30 min led by the primary investigator (H.C.), which provided an overview of the scoring tools and their interpretation with three examples on how to estimate and calculate mSWAT. This session included a review of the clinical characteristics of CTCL lesions, ensuring consistent recognition, differentiation, and classification across raters. Dermatology residents performed mSWAT assessments for each patient and these assessments were subsequently compared with mSWAT results conducted by an experienced co-author of the study (R.B.-B.). In the first step of the data analysis, descriptive statistics of research indicators, i.e., result ranges for individual patients (marked respectively P1–P14) assessed by all physicians were calculated (Table 1). To determine the shapes of the obtained distributions, statistics such as range (min-max), measures of central tendency (mean) and dispersion (standard deviation), measures of asymmetry and concentration (skewness, kurtosis), and normality tests of the distribution were calculated. In order to check whether the obtained distributions differed from the theoretical normal distribution, the Shapiro–Wilk tests were used, as suggested for relatively small sample sizes [14]. Additionally, patient P1 underwent a pilot High-frequency ultrasound (HFUS) assessment of the skin lesion (Figure 3). The study was conducted in accordance with the principles outlined in the Declaration of Helsinki and was approved by the Bioethics Committee at the Medical University of Wrocław. The approval code is KB–851/2021 and the approval date is 28 October 2021.

## 3. Results

### 3.1. Evaluation of Interrater Agreement and Interrater Reliability of mSWAT

The obtained statistical values indicated that in half of the cases, the variables did not show statistically significant differences from the normal distribution. The indicators for patients (P as patient and number without space) P2, P4, P7, P8, P10, P13, and P14 had distributions that differed from the normal one (the test result turned out to be statistically significant). The kurtosis statistics for the variables obtained for patients P2, P10, P13, and P14 indicated a clear platikurticity, i.e., a large dispersion of the results from the mean, while the variables for patients P4 and P7 were characterized by a clear concentration around the mean (leptokurticity of distributions). Due to limited sample sizes, we did not compare mSWAT scores between junior and senior residents. Additionally, patient P1 underwent a HFUS evaluation of the skin lesion (Figure 3), which revealed a narrow hypoechogenic band, measuring a maximum of 0.1 mm, located beneath the entrance zone (EZ).

### 3.2. Differences from the Reference Value

A reference value, a point referred to as a normal value, was set for each patient individually. Then, it was estimated whether the reference value significantly differed from the average results of the evaluators’ assessments. This was carried out separately for each patient. The Student’s *t*-test was used for one sample, while the comparisons were made for each patient separately. The results are summarized in Table 2.

The test result showed statistically significant differences from the reference (normal) value for 9 out of 14 patients (64.29%). Patients P1, P2, P4, P6, P8, P10, P11, P13, and P14 received significantly lower marks from the evaluating physicians than they should have (according to the correct reference value) (Table 2). For patients P3, P5, P7, P9, and P12, physician ratings were also lower than the reference value on average, but were not low enough to distinguish them from random differences for the measurement.

## 4. Discussion

A precise classification of skin lesion severity relies heavily on the subjective assessment of clinical manifestations by a physician, making the doctor’s experience crucial [15]. Assessing disease severity can be particularly challenging for young, inexperienced dermatologists. The choice of assessment method depends on whether it is intended for research purposes or routine clinical practice. Proper classification of the patient according to disease severity is essential for selecting an appropriate treatment modality. MF presents distinctively compared to other malignant skin tumors. In its early stages, MF often resembles inflammatory skin conditions, such as psoriasis or eczema, which frequently leads to misdiagnosis [16]. This clinical overlap makes it challenging to classify MF accurately within the traditional TNMB staging system. A key limitation of applying TNMB staging to MF lies in the objective assessment of body surface area (BSA) involvement. In early-stage MF, the classification system only distinguishes between lesions covering less than 10% (T1) and greater than 10% (T2) of the total BSA, failing to account for substantial differences in disease burden, such as between patients with 20% versus 50–70% BSA involvement. These limitations highlight the need for the development of new tools to assess disease severity more accurately in patients with MF/SS. The mSWAT is particularly advantageous for evaluating MF/SS, where various lesion types can co-exist within a single patient. It quantifies disease severity by recording the percentage of body surface area (BSA) affected by patch, papule/plaque, and tumor lesions. Tumors are defined as solid or nodular lesions exhibiting depth or vertical growth with a diameter of ≥1 cm. Each lesion type is assigned a relative weight—1 for patches, 2 for plaques, and 4 for tumors. These weighted values are then summed to produce a total score. BSA involvement is determined using the palmar surface of all five fingers (approximately 1% of BSA) (Figure 1). However, as indicated by both the literature and findings from our study, mSWAT has limitations as an assessment tool for MF/SS severity. It remains, to a significant extent, subjective and is influenced by the experience level of the evaluating physician. Furthermore, the weighting assigned to tumors in the mSWAT score has been debated due to the high prognostic value of these lesions. Tumors exhibit a much denser dermal infiltrate and greater neoplastic cell content than patches. Nonetheless, mSWAT may under-represent tumor burden relative to patches and plaques, complicating potential adjustments [12]. The high variability in classifying lesions as plaque or tumor complicates increasing the tumor weighting factor and highlights the need for a consistent assessor in clinical trials. The findings of this study align with our results, indicating statistically significant deviations from reference (normal) values in 9 of 14 patients (64.29%). Among the enrolled patients, only three presented with tumors (Figure 3 and Table 1 and Table 2). However, statistically significant discrepancies in assessments were observed in 2 of these 3 cases (P2 and P4), where tumors were misinterpreted as patches and plaques, leading to an underestimation of the mSWAT scores. The third patient with tumors (P3) also showed lower mSWAT scores, but these differences did not reach statistical significance. Assessment challenges were also evident in patients with erythroderma and infiltrative lesions. Specifically, for erythroderma in SS patients, P8 demonstrated statistically significant deviations in resident evaluations, while P7 showed lower mSWAT scores that, although not statistically significant, were noticeably distinct from random variations. This suggests resident physicians encountered difficulty in accurately evaluating mSWAT scores in erythrodermic presentations. Infiltrative skin lesions also posed additional challenges. All patients with infiltrative lesions (P6, P10, P13, and P14) demonstrated statistically significant underestimations in mSWAT scores by resident physicians. Interestingly, the highest-rated changes were observed in infiltrative and erythematous lesions of a single patient (P1), where statistically significant differences in mSWAT assessments were noted. For P5 and P12, mSWAT scores were lower but not statistically significant, whereas P11’s mSWAT scores were assessed accurately (Figure 3 and Table 1 and Table 2). These findings underscore the challenges resident physicians face in accurately evaluating mSWAT scores, particularly in complex presentations such as tumors, erythroderma, and infiltrative lesions. Although the currently available literature includes articles on mSWAT as a tool for assessing the severity of skin lesions in MF, highlighting its usefulness in monitoring disease progression and response to treatment [17], and publications providing information on the application of mSWAT for assessing skin tumor burden in patients with MF/SS [18], only 1 direct study comparing mSWAT assessments conducted by different physicians has been identified [19]. In 2007, Scarisbrick et al. [19] conducted a study to evaluate the variability in skin scoring among healthcare professionals. Physicians and nurses attended a 2-h lecture on skin scoring methods, combining the Rule of Nines and the Palmar method. Following the lecture, six patients with different stages of MF were assessed by both the lecturer and the attendees. A total of 22 attendees provided 122 scores, which were compared to the lecturer’s scores. For patient 1, the lecturer’s score was 73.6 compared to the attendees’ median of 50 (range: 26.4–99). For patient 2, the scores were 125.6 vs. 76.2 (range: 63–164), for patient 3, 50.7 vs. 57 (range: 17–91), for patient 4, 3.5 vs. 9.3 (range: 1.5–15.4), for patient 5, 4.7 vs. 15.9 (range: 4.2–25), and for patient 6, 17.3 vs. 17.2 (range: 14–28). Individual attendees showed consistent tendencies to score either above or below the lecturer’s assessments. The widest range of scores was observed for hypopigmented MF, while the least variability was noted in scoring erythroderma. Despite standardized training, significant interobserver variability in skin scoring was evident. This variability was partly attributed to differences in defining the palmar surface area (palm, fingers, and thumb) and its allocation of percentage surface area in different scoring systems.

Our study appears to be the second to address the topic of interobserver agreement in the use of mSWAT. Moreover, both our study and the similar study conducted by Scarisbrick et al. [19] demonstrated that mSWAT assessment is highly subjective and significantly influenced by the experience of the evaluating physician. However, the authors of the first study primarily focused on differences in the assessment of Body Surface Area (BSA) among the participating physicians, rather than on distinguishing the types of skin lesions in patients with CTCL.

The recent literature highlights that HFUS provides precise evaluation of infiltration depth and morphological characteristics of MF/SS lesions, offering valuable insights for the staging of CTCL. Moreover, the unique ultrasonographic features identified in MF and SS have the potential to support their diagnosis [20,21,22,23,24]. In a study by Wang et al. [20], 26 patients underwent HFUS examination. Among 16 patients with patches or plaques (early-stage group), a subepidermal low-echogenic band was identified, with only three plaque-stage lesions showing partial extension into the superficial dermis. Conversely, seven patients with tumors (advanced-stage group) exhibited lesions infiltrating the deep dermis or subcutaneous tissue. Significant differences were observed between early and advanced stages in terms of infiltration depth, boundary definition, and echo homogeneity. Furthermore, two folliculotropic MF lesions and one SS lesion displayed distinctive features, including a well-defined subepidermal low-echogenic band and patchy hypoechoic regions surrounding hair follicles within the dermis. Furthermore, in the study by Polańska et al. [21], the relationship between histopathological infiltrates containing clonal T cells and ultrasonographic findings was analyzed. HFUS performed on 10 patients with MF revealed the presence of a subepidermal low-echogenic band (SLEB). Notably, a strong correlation was identified between SLEB thickness and the thickness of subepidermal infiltration (r = 0.994, *p* < 0.05). The authors highlighted that SLEB is a characteristic feature of the infiltrative stage of MF, with its thickness potentially varying based on the type of skin lesion. The study suggests that HFUS may serve as a reliable noninvasive method for the quantitative assessment of MF, reflecting the extent of T-cell infiltration observed in histopathology. In another study by Polańska et al. [22], HFUS was utilized to objectively monitor the treatment response in three patients with MF over a 5-year period. The findings demonstrated a reduction in the thickness of the SLEB during phototherapy. The authors suggested that regular SLEB thickness measurements could provide a valuable supplementary method for monitoring therapeutic outcomes. Furthermore, the incomplete resolution of SLEB was associated with a lack of complete remission as assessed by mSWAT. Next, in a study conducted by Wohlmuth-Wieser et al. [23], epidermal thickness, SLEB thickness, and dermal thickness were measured and compared among patients with CTCL, atopic dermatitis (AD), and psoriasis. The mean epidermal thickness in CTCL patch lesions was 271 ± 124 µm. A subepidermal low-echogenic band (SLEB) was present in all CTCL patients, with grade 2 observed in 16.7% and grade 3 in 83.3% of cases. The mean SLEB thickness was 193 ± 78 µm, and the mean dermal thickness was 1847 ± 460 µm. Patients with CTCL and psoriasis showed a trend toward increased epidermal thickness compared to those with AD, suggesting a notable difference in epidermal characteristics between these conditions.

**Figure 4:**
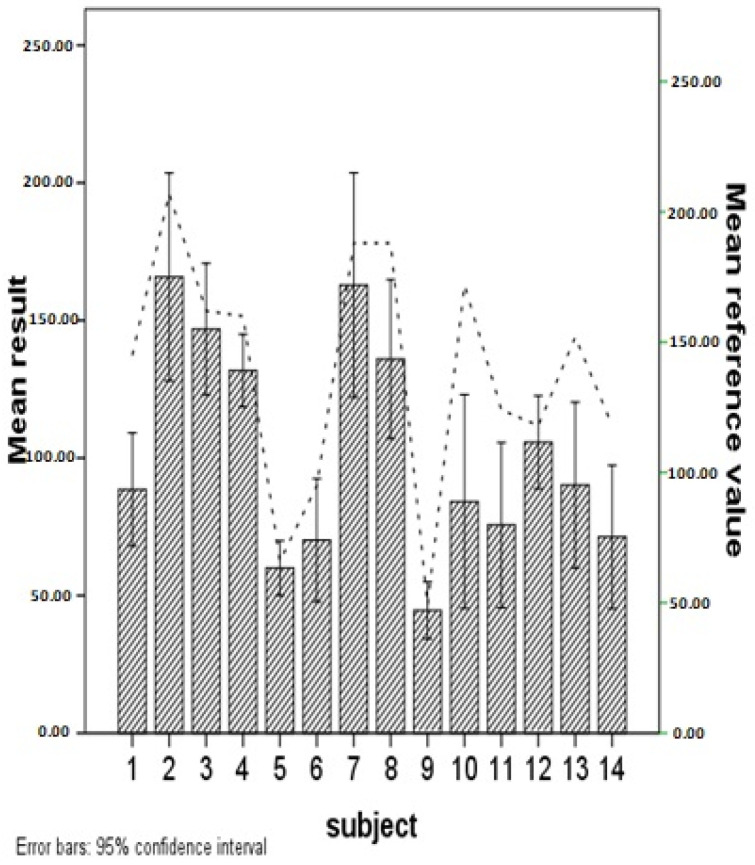
Differences between the mSWAT score carried out by residents compared to the reference value.

We suggest that, alongside mSWAT scoring, the HFUS examination might be performed to objectively monitor disease progression and treatment effectiveness in patients with MF/SS [24]. Thanks to this method the real infiltration could be estimated and counted in mm (Figure 4).

Moreover, other diagnostic tools, such as dermoscopy and reflectance confocal microscopy (RCM), may also be useful for diagnosis, assessment of disease severity, and monitoring remission of patients with CTCL. In the study by Soliman et al. [25], 88 MF patients were analyzed using H&E-stained sections, showing CD3, CD4, and CD8 positivity with CD7 negativity. Dermoscopy identified common features: non-homogeneous pink to erythematous background, orange discoloration, whitish scales, dotted and short linear vessels, and spermatozoa-like vessels, with variations across MF subtypes. In another study by Melhoranse Gouveia et al. [26], 38 MF lesions were analyzed by RCM, with 19 re-assessed by RCM after 6 months. In total, 50 biopsies were performed (38 at baseline, 12 at follow-up). An RCM checklist combining four features—Pautrier’s microabscess, epidermal and junctional lymphocytes, and interface dermatitis—predicted disease severity with statistical significance.

This study has several limitations. First, the small sample size of both patients and resident examiners may limit the generalizability of the findings. Second, patient photographs were utilized to standardize the assessment results while ensuring anonymity and respecting patient dignity. For legal and ethical reasons, facial and genital areas were not photographed, as shown in Figure 2. These constraints, although necessary for ethical and legal compliance, may have limited the comprehensiveness of visual assessments.

## 5. Conclusions

This study underscores the challenges and variability in the assessment of MF/SS using the mSWAT, particularly among less experienced resident physicians. Significant deviations from reference values were observed in 64.29% of patients, revealing the subjective nature of mSWAT scores and their sensitivity to the assessor’s expertise (Figure 3 and Table 1 and Table 2). Tumors and infiltrative lesions were identified as particularly challenging for resident physicians to evaluate accurately. Misclassification of tumors as patches or plaques in patients with advanced lesions led to substantial underestimation of mSWAT scores, demonstrating the difficulty in distinguishing between lesion types and the limitations of the current weighting system.

The findings highlight that erythrodermic and infiltrative lesions, especially in SS patients, also pose significant assessment challenges, with notable discrepancies in mSWAT scores among resident physicians for these cases. The interobserver variability evident in this study suggests a need for enhanced training and standardized assessment protocols to improve consistency and accuracy in mSWAT scoring, particularly for less experienced assessors.

Despite mSWAT’s utility in monitoring disease progression and treatment response, its limitations—such as the under-representation of tumor burden—indicate that it may not fully capture the complexity of MF/SS disease manifestations. This study is the second to address interobserver agreement in mSWAT use, pointing to the necessity of a more objective assessment tool as HFUS to monitor treatment in MF/SS patients (Figure 1, Figure 3 and Figure 4).

## Figures and Tables

**Table 1:** Descriptive statistics of research indicators: detailed assessments for patients (P1–P14), with participation from 16 residents in the study.

	**R**	**M**	**SD**	**Mdn**	**Sk**	**Kurt**	**W**
P1	9.00–145	88.56	38.60	81.00	−0.06	−0.47	0.91
P2	62.00–246	165.78	70.92	168.00	−0.14	−1.88	0.85 *
P3	53.00–212	146.81	44.89	160.00	−0.67	−0.26	0.93
P4	60.00–168	131.75	24.71	135.00	−1.48	4.14	0.87 *
P5	21.00–96	59.97	18.60	60.00	−0.10	0.17	0.98
P6	12.00–156	70.13	41.99	67.00	0.42	−0.59	0.95
P7	23.00–376	162.88	76.51	178.00	0.96	3.74	0.83 **
P8	21.00–188	135.94	54.26	159.00	−1.00	−0.42	0.83 **
P9	4.50–82	44.66	19.36	47.50	−0.26	0.41	0.97
P10	4.00–174	84.19	72.89	58.00	0.36	−1.85	0.78 **
P11	0.00–160	75.63	56.34	74.00	−0.01	−1.65	0.90
P12	39.00–160	105.75	31.75	112.00	−0.79	0.45	0.92
P13	18.00–152	90.13	56.62	92.00	−0.12	−1.93	0.81 **
P14	4.00–124	71.31	48.86	75.50	−0.24	−1.80	0.83 **

* *p* < 0.05; ** *p* < 0.01. Designations of abbreviations (in alphabetical order): Kurt—kurtosis of the distribution; M—mean; Mdn—median; P1–P14—patients 1–14; R—range; SD—standard deviation; Sk—skewness of the distribution; W—Shapiro–Wilk test.

**Table 2:** Differences between patient-by-patient mSWAT scores and the reference value.

	Ref	M	SD	t (15)	*p*
P1	145	88.56	38.60	−5.85	<0.001
P2	207	165.78	70.92	−2.33	0.035
P3	162	146.81	44.89	−1.35	0.196
P4	160	131.75	24.71	−4.57	<0.001
P5	66	59.97	18.60	−1.30	0.214
P6	95	70.13	41.99	−2.37	0.032
P7	188	162.88	76.51	−1.31	0.209
P8	188	135.94	54.26	−3.84	0.002
P9	49	44.66	19.36	−0.90	0.384
P10	172	84.19	72.89	−4.82	<0.001
P11	124	75.63	56.34	−3.44	0.004
P12	118	105.75	31.75	−1.54	0.144
P13	152	90.13	56.62	−4.37	0.001
P14	118	71.31	48.86	−3.82	0.002

Designations of abbreviations (in alphabetical order): M—mean; *p*—*p*-value/statistical significance of the test; ref—reference value (correct); P1–P14—patients 1–14; SD—standard deviation; t—Student’s *t*-test result.

## Data Availability

The data presented in this study are available on request from the corresponding author.
